# Cardiac arrest in pregnancy with successful stabilization and delivery on veno-arterial extracorporeal membrane oxygenation: a case report

**DOI:** 10.1093/ehjcr/ytae551

**Published:** 2024-10-17

**Authors:** Alice Burton, Seshika Ratwatte, David Zalcberg, Matthew Morgan, Rajit Narayan, Rachael Cordina

**Affiliations:** RPA Women and Babies, Royal Prince Alfred Hospital, Missenden Rd, Camperdown, NSW 2050, Australia; Department of Cardiology, Royal Prince Alfred Hospital, Missenden Rd, Camperdown, NSW 2050, Australia; Faculty of Medicine and Health, University of Sydney, Science Rd, Camperdown, NSW 2050, Australia; Faculty of Medicine and Health, University of Sydney, Science Rd, Camperdown, NSW 2050, Australia; Department of Anaesthetics, Royal Prince Alfred Hospital, Missenden Rd, Camperdown, NSW 2050, Australia; Intensive Care Services, Royal Prince Alfred Hospital, Missenden Rd, Camperdown, NSW 2050, Australia; Faculty of Medicine, Health and Human Sciences, Macquarie University, 75 Talavera Rd, Macquarie Park, NSW 2109, Australia; RPA Women and Babies, Royal Prince Alfred Hospital, Missenden Rd, Camperdown, NSW 2050, Australia; Department of Cardiology, Royal Prince Alfred Hospital, Missenden Rd, Camperdown, NSW 2050, Australia; Faculty of Medicine and Health, University of Sydney, Science Rd, Camperdown, NSW 2050, Australia

**Keywords:** Case report, ECMO, Pregnancy, Coronary aneurysm

## Abstract

**Background:**

Cardiac arrest in pregnancy is rare. Clinicians need to adapt management to the altered anatomy and physiology of pregnancy, and the well-being of two patients (mother and foetus) may come into consideration. The medical literature has limited reports on outcomes following extracorporeal membrane oxygenation (ECMO) in pregnancy.

**Case summary:**

We report the evaluation, management, and outcome of a woman with cardiac arrest and severe left ventricle (LV) dysfunction in mid-trimester of pregnancy. The previously well woman had tolerated two prior term pregnancies without complication. At 25 weeks of gestation, she presented to the hospital with breathlessness and vomiting after a pre-syncopal episode at home. She then had in-hospital cardiac arrest, managed initially with cardiopulmonary resuscitation. The LV was dilated, thin walled, and severely impaired (LV ejection fraction 14%), and there was a secundum atrial septal defect (ASD). She was supported with veno-arterial ECMO. Planned birth occurred 5 days post-arrest for maternal indication. Coronary angiography demonstrated 99% proximal left anterior descending artery stenosis and aneurysm, raising the possibility of previous subclinical Kawasaki disease. She underwent surgical revascularization and ASD closure. Both mother and infant made a good recovery.

**Discussion:**

We report a case of cardiac arrest in pregnancy as first presentation of severe LV dysfunction. The case highlights the role of ECMO for cardiac arrest in pregnancy and outlines specific interventions and management concepts in this setting.

**Learning points**

- To explore the technical aspects and supporting evidence for use of veno-arterial extracorporeal membrane oxygenation in pregnancy and the peripartum period
- To review advanced life support measures in cases of maternal cardiac arrest
- To evaluate a case of cardiac arrest in the setting of previously undiagnosed ischaemic cardiomyopathy and pregnancy

## Primary specialties involved other than cardiology

Obstetrics, Anaesthetics, Intensive care, Neonatology

## Introduction

Cardiac arrest in pregnancy is rare, affecting an estimated 1 in 36 000 pregnant women.^1^ Common causes of cardiac arrest in pregnancy include hypovolaemia, venous thromboembolic events, anaesthetic complications, amniotic fluid embolism, and cardiac conditions including myocardial infarction, sudden arrhythmia, and cardiomyopathy.^1^

The altered physiology of pregnancy affects not only presentation but also management options. Consideration of two patients (the mother and the foetus) can present additional challenges, especially in scenarios where delivery to facilitate maternal resuscitation may confer significant prematurity—and therefore morbidity—on the infant. Furthermore, there is a paucity of evidence to guide advanced life-saving interventions in pregnancy due to common exclusion of pregnant women from research.

Management of cardiac arrest in pregnancy includes conventional cardiopulmonary resuscitation (CPR, adapted to the altered anatomy and physiology with uterine displacement and/or perimortem caesarean, supplemental oxygen, and intubation with endotracheal tube) and vasopressors. In specialized care settings, extracorporeal membrane oxygenation (ECMO) during pregnancy may be an option following cardiac arrest. Case series data suggest a similar maternal safety profile for ECMO as in the non-pregnant population while there is a paucity of infant outcome data.^2^ Certain cardiac investigations and interventions (cardiac catheterization, electrophysiology studies, bypass, and transplant) are considered very high risk in pregnancy and are not commonly performed.

## Summary figure

Timeline of clinical events with representation of veno-arterial extracorporeal membrane oxygenation in pregnancy. CPR, cardiopulmonary resuscitation; VA-ECMO, veno-arterial extracorporeal membrane oxygenation; ASD, atrial septal defect; ICD, implantable cardiac defibrillator. Image created with BioRender.com.

**Figure:**
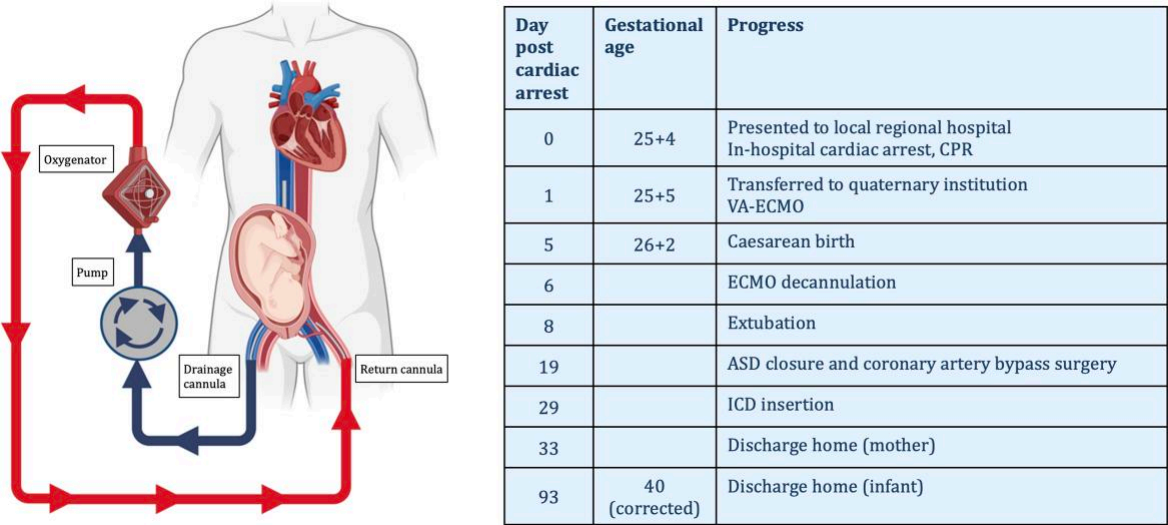


## Case presentation

**Figure 1:**
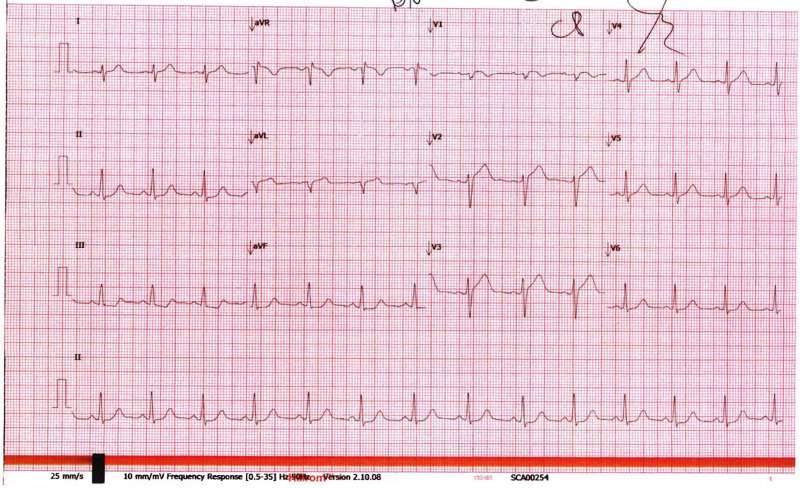
Electrocardiogram on initial presentation to emergency department showing sinus rhythm with no diagnostic ST segment changes of ischaemia.

**Figure 2:**
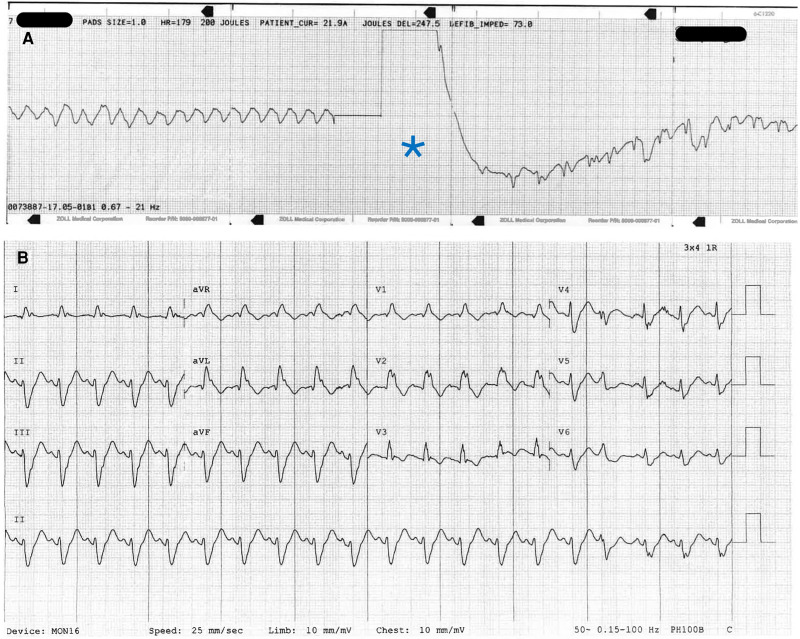
Electrocardiograms at time of cardiac arrest showing (*A*) coarse ventricular fibrillation and defibrillation shock (denoted by *) and (*B*) after return of spontaneous circulation showing right bundle branch block.

A 30-year-old multiparous Caucasian woman (gravida three, para two) presented to her local emergency department (regional centre) at 25 weeks and 4 days of gestation with breathlessness and vomiting after a pre-syncopal episode at home. Collateral history revealed several days of vomiting and diarrhoea. At triage, her blood pressure was 93/61 mmHg, heart rate was 87 b.p.m., respiratory rate was 20 breaths per minute, and oxygen saturations were 99% on room air. Initial electrocardiogram demonstrated sinus rhythm with no ST segment changes suggestive of ischaemia (*Figure 1*). The patient did not have any significant medical, surgical, or social history, and two prior pregnancies had been uncomplicated. She was a non-smoker with no substance use, taking pregnancy multi-vitamins as the only medications. The patient had a cardiac arrest in the emergency department; the initial rhythm was ventricular fibrillation (*Figure 2A*). Resuscitation was commenced according to advanced life support (ALS) guidelines and continued for 8 min before return of spontaneous circulation (ROSC; *Figure 2B*). The patient was intubated and transferred to our quaternary centre for specialized multidisciplinary team (MDT) support including obstetrics, maternal cardiology, neonatology, and an intensive care with ECMO capabilities.

**Figure 3:**
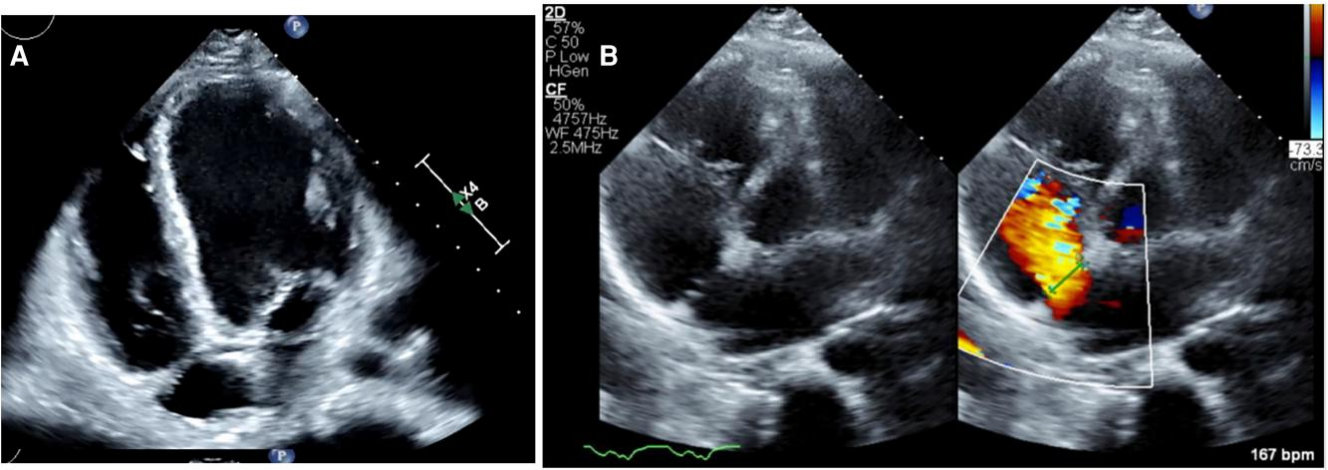
Transthoracic echocardiogram prior to commencing veno-arterial extracorporeal membrane oxygenation demonstrating (*A*) dilated left ventricle with thinned septum and apex on apical four-chamber view and (*B*) secundum atrial septal defect with left-to-right shunting with colour Doppler on parasternal short-axis view.

**Figure 4:**
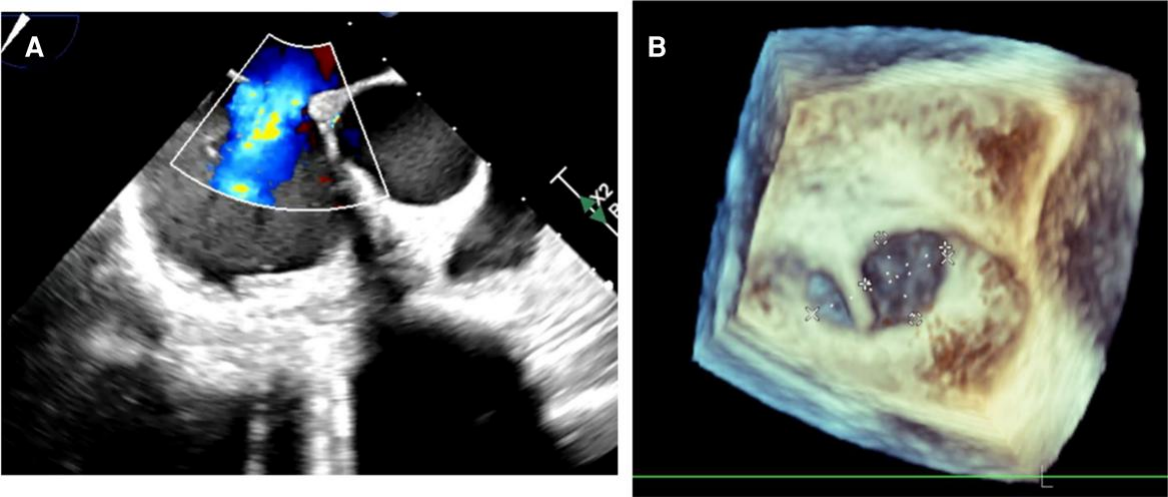
Transoesophageal echocardiogram showing (*A*) two-dimensional image of secundum atrial septal defect with left-to-right shunt and (*B*) three-dimensional reconstruction of atrial septal defect measuring 16 × 8 mm with muscular band separating two sides.

On arrival to our centre, the cardiopulmonary examination was remarkable for tachycardia (140 b.p.m.) and narrow pulse pressure. Electrocardiogram showed sinus tachycardia with right bundle branch block and no dynamic changes on serial assessment. Peak troponin was 2688 ng/L (normal < 14 ng/L), also non-dynamic, and NT-proBNP was 3632 ng/L (normal <125 ng/L). Transthoracic echocardiogram (TTE) showed a dilated, thin walled, left ventricle (LV) with severe global impairment [LV ejection fraction (LVEF) 14%; *Figure 3A*]. The right ventricle was dilated and moderately impaired. A 16 mm secundum atrial septal defect (ASD) was noted with left-to-right shunt on colour Doppler (*Figure 3B*) and confirmed with transoesophageal echocardiography. It was not anatomically suitable for percutaneous closure due to a band of tissue dividing the defect (*Figure 4*). We suspected a chronic cardiomyopathy rather than an acute ischaemic event (due to LV wall thinning) and a concurrent congenital ASD. Bedside obstetric ultrasound demonstrated profound foetal bradycardia (65 b.p.m.) with absent umbilical artery end-diastolic flow (a sign of significant foetal compromise).

The patient was assessed to be in cardiogenic shock and was placed on veno-arterial (VA)-ECMO after collaborative team discussions. Her ASD acted as a natural vent; she continued ejecting while on mechanical support. A levosimendan infusion was given over 24 h, and intramuscular betamethasone was administered for foetal lung maturation. Anticoagulation was by heparin infusion, targeting anti-Xa level 0.3–0.5 IU/mL. At this point, MDT discussions determined that there were not maternal or foetal indications for delivery. For the mother, it was judged that the acute profound haemodynamic changes of birth could overwhelm her very limited cardiac reserve and that stabilization on ECMO was a better short-term goal. For the foetus, it was considered that immediate delivery and the morbidity of extreme prematurity would only compound the already poor prognosis.

Over the following 4 days on VA-ECMO support, there was some improvement in LV function (LVEF 35%). The foetal condition improved with normalization of the foetal heart rate and umbilical artery Doppler waveform. The question of delivery was carefully deliberated, and the family were extensively counselled. Risks of prolonged ECMO were weighed against the foetal benefits of advancing the gestation. It was decided that delivery should be undertaken to reduce the cardiovascular demand and to facilitate more durable ventricular support such as ventricular assist device or cardiac transplant if required. Planned delivery occurred day 5 post-arrest (Day 4 of VA-ECMO and 26 weeks and 2 days of gestation).

In preparation for birth, anticoagulation was ceased 6 h pre-operatively with an activated partial thromboplastin time to confirm a return of baseline coagulation. Magnesium sulfate (standard concentration) infusion was commenced 4 h prior to birth for foetal neuroprotection. The patient remained intubated and on VA-ECMO support. Anaesthesia for caesarean delivery included a propofol infusion (target control infusion 2 mcg/mL), fentanyl infusion (60 mcg/h), rocuronium boluses every 30 min, and a noradrenaline infusion (titrated to mean arterial pressure > 65 mmHg). A tranexamic acid load of 1 g was also given to prevent hyperfibrinolysis in the event of a postpartum haemorrhage. A single unit of oxytocin was given at the time of delivery followed by a bolus of vasopressor to counteract the vasodilatory effects of oxytocin. Caesarean delivery was performed by classical uterine incision. Birth weight (780 g) was age-appropriate; Apgar scores were 5, 6 and 7 at 1, 5, and 10 min, respectively; and the umbilical artery blood pH was normal. Estimated blood loss was 500 mL, and two units of packed red blood cells were administered.

**Figure 5:**
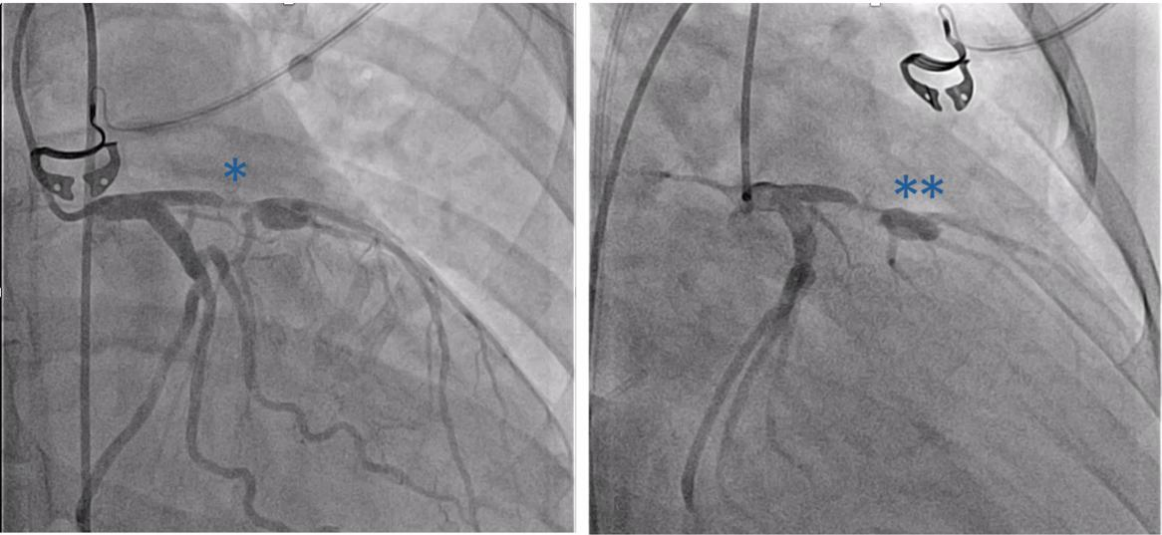
Coronary angiogram showing 99% left anterior descending artery stenosis (*) immediately proximal to a large left anterior descending artery aneurysm (**).

**Figure 6:**
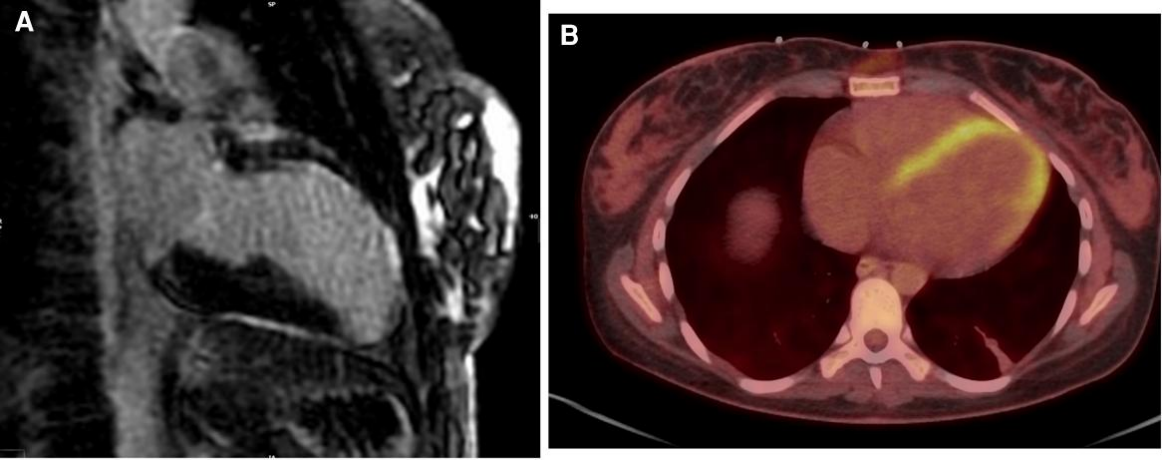
Tissue viability studies. (*A*) Cardiac magnetic resonance imaging scan showing extensive, late gadolinium enhancement primarily affecting the anterior, anteroseptal, and apical segments. (*B*) Positron emission tomography scan showing global reduction (but not absence) of glucose metabolism in the left ventricle.

The patient was successfully decannulated from VA-ECMO the day after delivery and then extubated 2 days later (Days 6 and 8 post-arrest, respectively). Further cardiac investigations were undertaken. Coronary angiography demonstrated 99% proximal left anterior descending (LAD) artery stenosis, immediately proximal to a large LAD artery aneurysm (*Figure 5*). Cardiac magnetic resonance imaging showed extensive delayed gadolinium enhancement primarily affecting the anterior, anteroseptal, and apical segments, suggesting a non-viable LAD territory (*Figure 6A*). Given the critical nature of the decision to revascularize, a positron emission tomography scan was used as a second modality to assess viability. This showed global reduction of glucose metabolism in the LV but viable myocardium (*Figure 6B*).

At Day 19 post-arrest, the patient underwent surgical revascularization and ASD closure, with a single left internal mammary artery graft used to bypass the LAD stenosis and aneurysm. Given her LV scarring and documented arrhythmia, a secondary prevention single-chamber implantable cardioverter defibrillator was implanted prior to discharge. She was commenced on cardioprotective medications including bisoprolol, sacubitril/valsartan, dapagliflozin, and aspirin. She elected not to breastfeed. The patient was discharged 1 month after initial cardiac arrest with repeat TTE showing that her LVEF had improved to 43%.

The infant faced typical challenges of extreme prematurity including jaundice, chronic lung disease (not requiring oxygen support by time of discharge), and retinopathy of prematurity (Stage II). She was discharged home at close to 40 weeks of corrected gestational age. Neuroimaging was normal.

At 12-month follow-up, our patient has had no further episodes of decompensated heart failure and did not sustain any long-term neurologic deficits from her initial arrest. Follow-up TTE shows a mildly impaired LVEF (45%) with no evidence of pulmonary hypertension. Current medications include low-dose aspirin, bisoprolol, and sacubitril with valsartan. Our patient has ongoing anxiety and post-traumatic stress following this event and is working with our service’s psychologist. The infant has met developmental milestones, and follow-up has so far predicted low chance of cerebral palsy.

## Discussion

Maternal cardiac arrest is an uncommon clinical occurrence. The frequency of cardiogenic cases may be expected to rise in settings with increasing survival from congenital and childhood cardiac disease and rising rates of advanced maternal age and obesity. Understanding the altered physiology and anatomy of pregnancy allows clinicians to effectively adapt resuscitation measures to this population.

Relevant changes in pregnancy include aortocaval compression from the supine-positioned gravid uterus which reduces cardiac output. Decreased systemic vascular resistance reduces arterial blood pressure and cardiovascular reserve while the compensatory increase in heart rate increases metabolic requirements. There is increased oxygen consumption in pregnancy, and lung compression by the expanding abdomen reduces functional residual capacity, contributing to quicker onset of hypoxaemia and more difficult ventilation. Breast tissue can hinder cardiac compressions while laryngeal oedema and oesophageal sphincter relaxation can hinder intubation.^3,4^

British and American ALS guidelines for in-hospital cardiac arrest highlight the following key modifications for pregnancy.^4,5^ Beyond 20 weeks of gestation, aortocaval compression should be alleviated by manual leftward displacement of the uterus and a left-lateral tilt while still maintaining effective compressions. Prompt and expert airway management should be prioritized (given the limited oxygen reserve and potentially difficult endotracheal intubation), and techniques to limit regurgitation are considered of lesser importance. Defibrillation, arrhythmia, and vasopressor medications are given at standard doses. Other suggestions include a specific maternal cardiac arrest team notification system and removal of any foetal monitoring. Simulation training may improve clinician competence and confidence^6^ while validated clinician questionnaires may assist in identifying training needs.^7^

Perimortem caesarean delivery is recommended to be performed if ROSC is not achieved by 4 min.^4,8^ The rationale is to assist maternal resuscitation by alleviating aortocaval compression and improving venous return, with the secondary goal of limiting infant neurological damage. A decision-to-birth interval of 1 min is recommended, though the likelihood of achieving such a target may be low.^9^

In reality, decisions around perimortem caesarean are complex and case-specific.^10,11^ For our patient, the initial cardiac arrest and resuscitation occurred in a regional setting without on-site surgical or neonatal resources. Focussing on effective CPR, defibrillation, and vasopressor support enabled the mother–foetus pair to be stabilized ahead of more definitive management and likely contributed to the good outcomes for both patients.

On arrival at our institution, it was judged that delivery would hinder rather than improve the ongoing maternal resuscitation. At caesarean, birth is associated with marked, acute increase in maternal heart rate and cardiac output, and sudden reduction in mean arterial blood pressure.^12^ These changes appear even more profound with administration of oxytocic agents (which is standard practice for preventing postpartum haemorrhage). There was concern that with such severe LV dysfunction, the patient would be unable to compensate for these haemodynamic shifts.

In this case, VA-ECMO provided circulatory and respiratory support post cardiac arrest. The published literature reporting use of VA-ECMO in pregnancy is comprised of case reports and series and small retrospective observational studies (there is greater experience with venovenous ECMO). In a systematic review that summarized these reports, the main indications for VA-ECMO in pregnancy were cardiac arrest, cardiac failure, and peripartum cardiomyopathy.^2^ Maternal survival was 72% (and 88% in women cannulated for cardiac arrest). These rates exceed those reported in the general population, likely reflecting the younger and healthier profile of pregnant women and the higher frequency of acute, reversible indications for ECMO (as well as potential publication bias). Additionally, rates of thrombotic and bleeding complications were similar to those reported in the general population.

Foetal and neonatal outcomes of ECMO in pregnancy are sparsely reported, and rates of preterm birth and neonatal intensive care unit (NICU) admission appear high.^2^ There are no data on long-term infant outcomes. In our case, the foetal condition significantly improved post VA-ECMO cannulation. While this is a logical sequela (better maternal circulation improving placental perfusion), we also speculate that the uterus and placenta are anatomically advantaged within the VA-ECMO circuit. Oxygenated blood is pumped retrograde from the femoral artery cannulation site. The proximity of the ipsilateral uterine artery (a branch of the anterior division of the internal iliac artery) makes it likely that a high oxygen concentration is delivered to the uteroplacental unit.

The period of VA-ECMO support also allowed foetal condition to be optimized ahead of very preterm delivery. At early gestation, corticosteroids, magnesium sulfate, and a further 5 days of gestation are all associated with better neonatal outcomes.^13^ Access to quaternary NICU was also important to the good outcomes in this case.

The cause of LAD aneurysm in this case is unknown. Around half of LAD aneurysms are attributed to atherosclerotic disease; less common causes include inflammatory, infectious and connective tissue disorders, and congenital lesions.^14^ In this patient, subclinical childhood Kawasaki disease was considered most likely due to the absence of generalized atherosclerotic disease and the angiographic appearance. It is possible that the gastrointestinal symptoms preceding her initial presentation precipitated a ventricular tachycardia storm at the time of arrest. Of interest, the patient had tolerated two previous pregnancies without complication. We postulate that her ASD provided a way to offload both LA and LV pressures, reducing back pressure into the pulmonary vasculature and thereby lowering the risk of pulmonary oedema in the setting of her poor left ventricular function.

## Conclusion

The increased cardiac demands of pregnancy may unmask underlying cardiac disease in women of reproductive age. Although rare, CPR and mechanical support may be required, with adaptation to the altered anatomy and physiology. Veno-arterial ECMO can be utilized to support women post cardiac arrest in pregnancy and advance the gestation (allowing stabilization of mother and infant ahead of birth). Multidisciplinary care underpins the management of such complex cases.

## Lead author biography


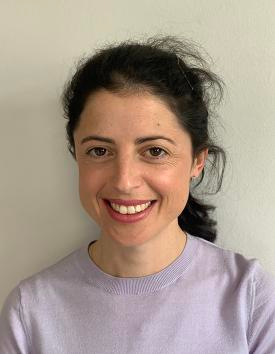


Alice Burton is an obstetrician and gynaecologist undertaking subspeciality training in maternal and foetal medicine. She has an interest in supporting women with congenital and acquired heart disease through pregnancy.

## Data Availability

Non-identifiable data underlying this article will be shared on reasonable request to the corresponding author.

## Notes

**Consent:** The authors confirm that written consent for submission and publication of this case report including the images and associated text has been obtained from the patient in line with the COPE guidelines.

**Funding:** S.R. is supported by the National Heart Foundation of Australia (grant 106796).
